# A Soft Sensor-Based Three-Dimensional (3-D) Finger Motion Measurement System

**DOI:** 10.3390/s17020420

**Published:** 2017-02-22

**Authors:** Wookeun Park, Kyongkwan Ro, Suin Kim, Joonbum Bae

**Affiliations:** Department of Mechanical Engineering, Ulsan National Institute of Science and Technology (UNIST), Ulsan 44919, Korea; wkpark@unist.ac.kr (W.P.); norudrhks@unist.ac.kr (K.R.); suinkim@unist.ac.kr (S.K.)

**Keywords:** soft sensor, wearable sensor, finger motion measurement, finger joint modeling

## Abstract

In this study, a soft sensor-based three-dimensional (3-D) finger motion measurement system is proposed. The sensors, made of the soft material Ecoflex, comprise embedded microchannels filled with a conductive liquid metal (EGaln). The superior elasticity, light weight, and sensitivity of soft sensors allows them to be embedded in environments in which conventional sensors cannot. Complicated finger joints, such as the carpometacarpal (CMC) joint of the thumb are modeled to specify the location of the sensors. Algorithms to decouple the signals from soft sensors are proposed to extract the pure flexion, extension, abduction, and adduction joint angles. The performance of the proposed system and algorithms are verified by comparison with a camera-based motion capture system.

## 1. Introduction

Owing to the increasing demand for virtual reality (VR) and augmented reality (AR), wearable systems that can easily measure human motion have been actively researched [1,2,3,4,5,6,7]. Accurate measurement of human motion is important for active interaction with a virtual environment. In particular, the measurement of finger movements, which play a crucial role in manipulating objects and interacting with the external environment, has become a key area of research for the realization of immersive interaction in VR and AR applications.

**Figure 1:**
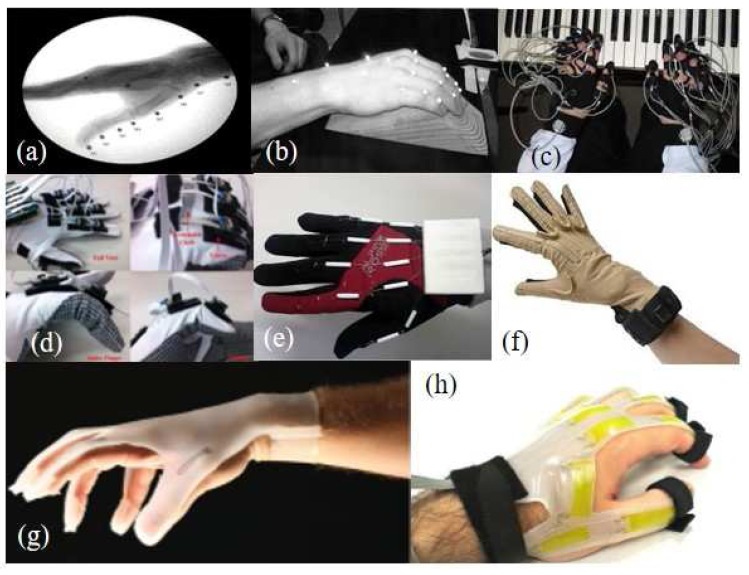
Examples of finger motion measurement systems: (**a**) X-ray based system [2]; (**b**) Infrared (IR) camera based system [3]; (**c**) Three dimensional position sensor system [4]; (**d**) Optical linear encoder based system [5]; (**e**) Potentiometer-based system [6]; (**f**) CyberGlove [7]; (**g**) A glove with embedded strain sensors [8]; (**h**) Wearable soft artificial skin [9].

Research efforts to measure finger motion have been significant. The system developed can be classified as glove and non-glove types. The non-glove systems shown in Figure 1a,b typically use multiple cameras with the reflective markers or X-ray to capture the human motion [2,3]. However, mobility is limited by the fact that the cameras are fixed, and by the requirement for peripherals.

In contrast, glove based measurement systems are more mobile. Magnetic sensors, optical linear encoders (OLE), potentiometers and flex sensors are used in glove-based systems as illustrated in Figure 1c–f [4,5,6,7]. Although these systems are able to measure finger joint angles quite accurately, the natural finger motion is restricted by the sensors to the glove. Even though some systems are able to measure 3-D finger motion, but these systems may have disadvantages in complexity of the system and accuracy of the measurement. The three dimensional magnetic sensor system, shown in Figure 1c, needs extra module to measure the position of the sensor, which makes the system complicated and bulky [4]. Cyberglove, shown in Figure 1f, measures the finger joint angles based on the modified anatomical model [7]. But, the modified model has many single degrees of freedom joints, and the distance between adjacent joints are widely varied depending on the individuals, which may cause inaccurate motion measurement for the different size of the hand.

Soft sensors have been used as an alternative method for measuring finger motion [8,9,10,11,12]. Soft sensors are typically fabricated from soft materials such as silicone, with embedded microchannels that are filled with a conductive liquid, such as EGaIn [13]. When an external force is applied to the sensor, the microchannels are deformed, creating a change in the electrical resistance of the conductive liquid. By measuring the change in resistance, the applied force, or strain of the sensor can be measured. As soft sensors use an embedded structure, the overall size of the system can be reduced. Also, the elasticity of soft materials reduces interference with the natural movement of the fingers.

Several systems have utilized soft sensors, as shown in Figure 1g,h [8,9]. However, only flexion/extension of the finger joints can be measured, and therefore complex finger joints with more than two degrees of freedom (DOF) cannot be represented. This is due in part to a lack of research into suitable positioning or the number of attached soft sensors based on the skeletal structure of the finger joints, and the extraction of flexion/extension and abduction/adduction motions from the sensor signals.

In this paper, a soft sensor-based 3-D motion measurement system is proposed. Also, modeling of the complex finger joints is presented, including the carpometacarpal (CMC) joint of the thumb, and the metacarpal (MCP) joint of the four fingers, which allow simultaneous measurement of the flexion/extension and abduction/adduction of joints. In addition, an algorithm for decoupling of the sensor signals is proposed for the measurement of 3-D motion. The performance of the proposed soft sensor-based 3-D finger motion system was verified by experiments with a camera-based motion capture (MoCap) system, Optitrack [14].

This paper is organized as follows. In Section 2, the configuration of the soft sensor-based finger motion measurement system is introduced. In Section 3, models of the complex finger joints are presensted, and the decoupling algorithm for the sensor signals is proposed in Section 4. In Section 5, the comparison of the joint angles and the fingertip position measurement is verified. Finally, conclusion and future work are given in Section 6.

## 2. Configuration of the Soft Sensor-Based 3D Finger Motion Measurement System

**Figure 2:**
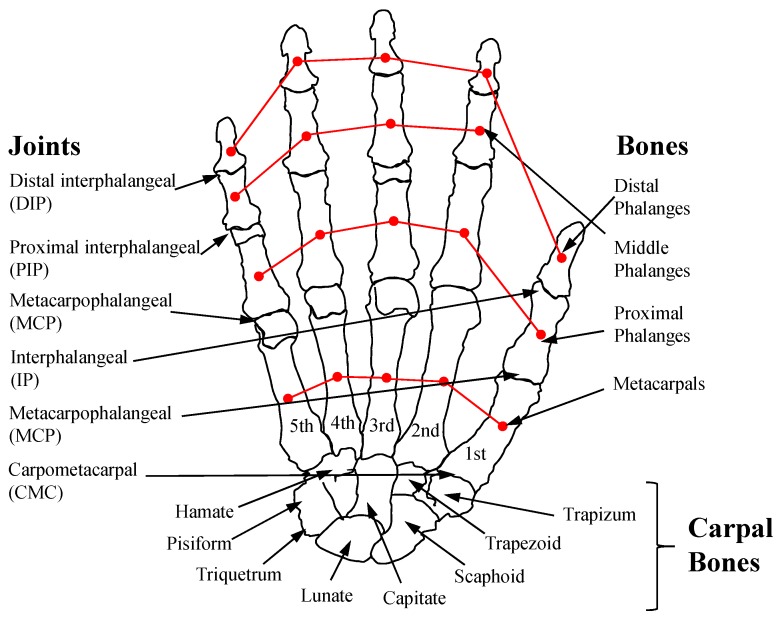
Skeletal structure of the human hand.

The finger motions are usually expressed by three joints: the MCP, proximal interphalangeal (PIP) and distal interphalangeal (DIP) joints (the CMC, MCP and IP joint for the thumb), as shown in Figure 2. The PIP and DIP joints each have a single DOF, but the MCP joint is typically modeled as universal joint with two DOFs. Owing to the musculoskeletal dependency between the PIP and DIP joints [15], the DIP joint angle can be estimated by measurement of the PIP joint angle. In the thumb, the MCP and interphalangeal (IP) joints are similar to the MCP and PIP joints of the other four fingers, but the CMC joint moves in complex and unique ways. Therefore, a simple and accurate model of the finger joint, especially the CMC joint, is important for the measurement of 3-D finger motions. The detailed modeling of finger joints will be discussed in Section 3.

**Figure 3:**
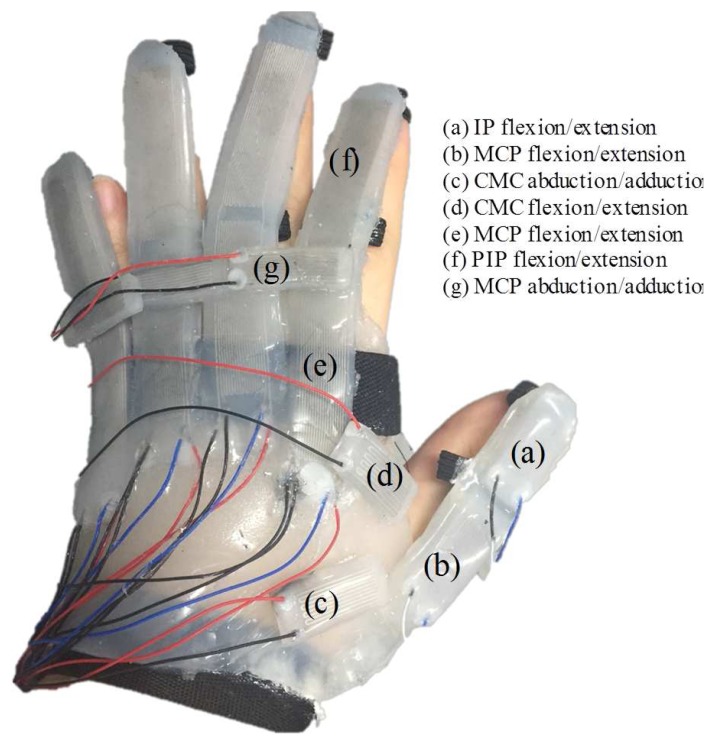
Configuration of the soft sensor-based 3-D finger motion measurement system.

**Figure 4:**
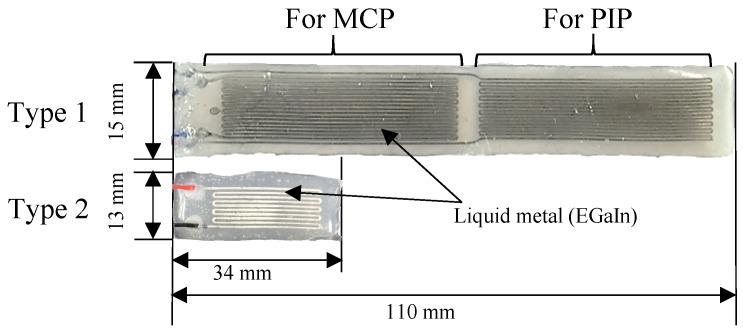
Prototypes of the soft sensors.

Considering the musculoskeletal structure of the hand, the proposed soft sensor-based finger motions measurement system was designed and fabricated as shown in Figure 3. A single sensor with two sensing regions (hereafter referred to as Type 1 sensor, shown in Figure 4), was used to measure the flexion/extension angles of the MCP, PIP and DIP joints. A short sensor (hereafter referred to as Type 2 sensor, shown in Figure 4) was designed to fit between the fingers for measurement of the abduction/adduction angles of the MCP joints. Type 2 sensors were also used to measure the motion of the thumb, due to its short length.

Type 1 sensors were attached to the index, middle, ring and little fingers (as shown in Figure 3e,f). Two microchannels in Type 1 sensor covered the MCP and PIP joints. The abduction/adduction motion of the MCP joint was measured by Type 2 sensor positioned between the phalanges of adjacent fingers without restricting natural movement (as shown in Figure 3g). To measure the CMC joint angle, two Type 2 sensors were used (as shown in Figure 3c,d). Type 2 sensors were also attached to the thumb to measure the flexion/extension of the MCP and IP joint (as shown in Figure 3a,b). The attached location of the sensors based on the finger model will be detailed in Section 3 and Section 4.

### 2.1. Design of the Soft Sensors

The soft materials used to fabricate the sensor shown in Figure 3 are flexible, and can be stretched and bent. Microchannels were molded into the soft structure, and filled with the conductive liquid metal, EGaIn [13]. Deformation of the microchannel changes the resistivity of the liquid metal [10,11]. The characteristics of the sensors, including the sensitivity and range of measurable strain, depend on the geometric parameters of the sensor body and the microchannel. Specifically, the microchannel narrows when stretched by an external load, which increases the resistance of the liquid between the both ends. Therefore, the expected resistance change was derived from the geometric parameters and fracture mechanics [12]:

(1) $$\Delta R=\frac{\rho \varepsilon L(8-\varepsilon )}{wh{\left(2-\varepsilon \right)}^{2}}$$

where ΔR is the change in resistance, in ohms [Ω], *ρ* is the electrical resistivity of the EGaIn, $29.4\times 10^{-2}$ [Ω·m], *ε* is the strain, *L* is the length of the microchannel in millimeters (mm), and *w* and *h* are the respective width and height of the microchannel in millimeters (mm).

**Figure 5:**
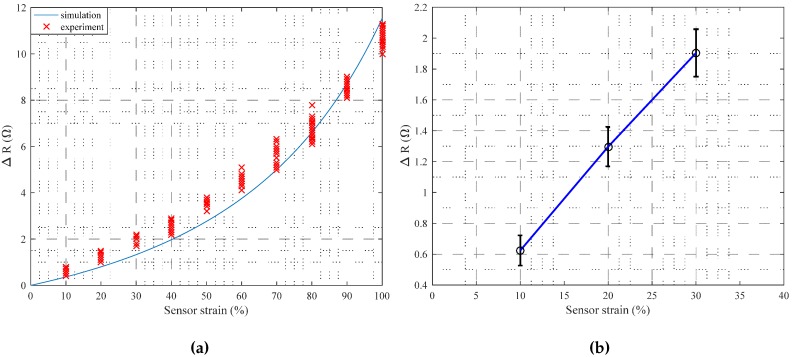
Resistance change of the soft sensor with respect to the strain; (**a**) 10%∼100% strain; (**b**) 10%∼30% strain.

As described in (1), the width and height of the microchannel determines the change in resistance ΔR for a given change in length *ε*. The actual performance of the fabricated soft sensor was verified by experiments. Aa shown in Figure 5a, the resistance change was measured from 10%∼100% (10% intervals, 25 trials). The experimental results were quite well-matched with the simulation; the error between the experiment and simulation may be caused by the dimensional error of the fabricated microchannel, etc. As shown in this figure, the sensor strain and the resistance change shows nonlinear relationship.

For a simple calibration process, the relationship between the resistance change and the strain needs to be linear. To ensure the linear property, the sensing units were designed to be stretched less than 30% on the skin, i.e., the microchannel was designed to have meaningful results within 30% of elongation. Up to 30% of the strain, the relationship is linear as shown in Figure 5b (blue line). The means (black dots) and ±standard deviations (black lines) of the resistance change for 10%, 20% and 30% of strain were displayed in the figure. The mean error of the resistance change by the linear approximation was about 0.126 Ω, which was about 3.67% of strain. Therefore, to maximize sensitivity and maintain feasible fabrication, the width and the height of the Type 1 and Type 2 sensors were determined to be 0.3 mm, 0.3 mm, and 0.5 mm, 0.5 mm, respectively. The geometric parameters are specified in Table 1, and two types of soft sensors were designed and fabricated, as shown in Figure 4.

### 2.2. Fabrication

Silicone (EcoFlex) [16] was used as the base material in the sensor and EGaIn was used as the electrically conductive liquid metal. The fabrication steps are similar to those described in [12].

First of all, the liquid silicone mix was poured into a mold, which had been fabricated using a computer numerical controlled (CNC) milling machine. The silicone poured in the mold was cured at 60 °C. Secondly, a base layer was formed by spin coating the liquid silicone on an acrylic plate at 400 rpm and cured at 60 °C. To bond these parts, the base layer was spin-coated with liquid silicone at 1800 rpm as an adhesive role. After that, the microchannel was brought into contact with the base layer, which contains the spin-coated liquid silicone. The bonded microchannel was cured at 60 °C for 30 min. After removing the unnecessary parts, a syringe was used to inject EGaIn into one end of the microchannel. Simultaneously, the air in the microchannel was evacuated using a second syringe at the opposite end. After injection, wires were connected through the holes made by the syringes, and the holes were filled with silicone to seal the channel to ensure the EGaIn do not spill out. Two completed sensors are shown in Figure 4.

## 3. Modeling of Finger Joints

### 3.1. Modeling of Four Fingers

Basically, the soft sensors were designed to measure linear displacement as the finger motions can be converted to linear displacement above the finger joints. Based on this, complex finger joints were modeled and the models were used to determine the sensor positions required to measure the complex finger motion, and extract joint angles.

**Figure 6:**
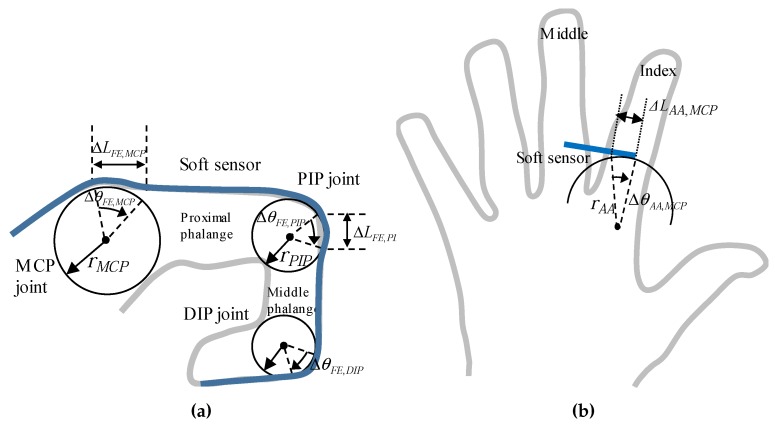
Modeling of the metacarpophalangeal (MCP) and the proximal interphalangeal (PIP) joints; (**a**) Flexion/extension; (**b**) Abduction/adduction.

The PIP and DIP joints have a single DOF, and therefore do not need complicated models. Also, the MCP joint was typically modeled as a universal joint with two DOFs, including the flexion/extension and abduction/adduction motions. The location of the soft sensor was determined by the rotation axis based on the models. Thus, the positioning of the soft sensors at the MCP, and PIP joints is obvious as shown in Figure 6a.

For the flexion/extension of the MCP, PIP and DIP joints, cylindrical models, which mean the change in circular arc was converted into the angle, were applied as shown in Figure 6a. The length change of the sensing unit at the MCP and PIP joints (Δ*L_FE,MCP_* and Δ*L_FE,PIP_*, respectively), were converted to the joint angle through a biomechanical model that was applied to calculate tendon excursions in the extensor muscles [15]. The flexion/extension angles of the MCP and PIP joints were calculated assuming that each joint defines a circle with radii of $r_{MCP}$ and $r_{PIP}$, respectively. Please note that the DIP joint can be modeled similarly with the MCP and DIP joints as shown in Figure 6a, but the DIP joint angle was not directly measured because the DIP joint angle can be estimated by the PIP joint angle due to the musculoskeletal dependency [15]. As a result, the linear displacement on the skin at the MCP and PIP joints were converted with $\theta_{FE,MCP}$ and $\theta_{FE,PIP}$, and the DIP joint angle was calculated from the PIP joint angle, as follows:

(2) $$\begin{array}{ccc} \Delta \theta_{FE,MCP} & = & \frac{\Delta L_{FE,MCP}}{r_{MCP}} \end{array}$$

(3) $$\begin{array}{ccc} \Delta \theta_{FE,PIP} & = & \frac{\Delta L_{FE,PIP}}{r_{PIP}} \end{array}$$

(4) $$\begin{array}{ccc} \Delta \theta_{FE,DIP} & = & \frac{2}{3}\Delta \theta_{FE,PIP} \end{array}$$

As shown in Figure 6b, the abduction/adduction motion was modeled as an arc trajectory between the fingers, which pivots on a central point between the capitate and trapezoid, shown in Figure 2. Because the range of the angle was relatively small, the angle was measured through the measurement of the linear displacement using (5).

(5) $$\begin{array}{ccc} \Delta \theta_{AA,MCP} & \approx & \frac{\Delta L_{AA}}{r_{AA}} \end{array}$$

As a result, Type 1 sensors were attached on each phalanges except for the thumb, for the flexion/extension of the four fingers. Type 2 sensor was attached between the adjacent proximal phalanges to measure the abduction/adduction angles.

Please note that the exact $r_{MCP}$ and $r_{PIP}$ were not needed since the strain of the soft sensor could be directly mapped with the joint angles. Namely, the linear relationship between the joint angles and the linear displacement on the joint were obtained experimentally from the calibration process, which will be discussed in Section 5, and the joint angles were calculated linearly with the strain of the soft sensor. Thus, the joint radii were only used to derive the relationship in (2)∼(5), but they were not actually masured and used in the system.

### 3.2. Modeling of the Thumb

**Figure 7:**
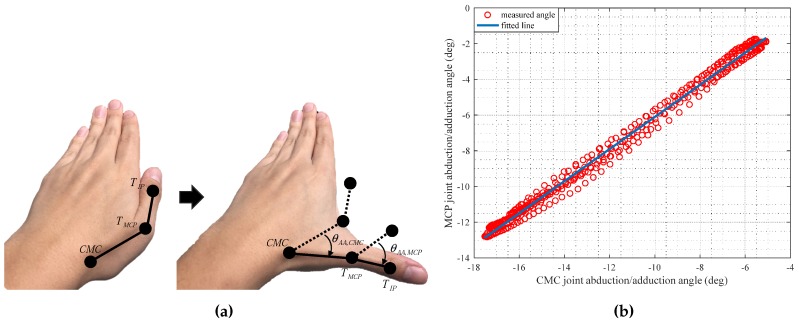
The relationship between the abduction/adduction of the carpometacarpal (CMC) and MCP joints; (**a**) Abduction/adduction of the CMC and MCP joints of the thumb; (**b**) Linear relationship between the CMC and MCP joint angles.

The MCP and IP joints of the thumb have similar models used for the four fingers shown in Figure 6a. As the IP joint is formed on the proximal phalanx, its shape is similar with the PIP joint of the four fingers [17]. The MCP joint of the thumb is known to have flexion/extension as well as abduction/adduction [18]. As shown in Figure 7a, while the thumb CMC joint was moved in a pure abduction/adduction from normal position, the abduction/adduction of the MCP joint is highly correlated with the abduction/adduction of the CMC joint because the two joints share a musculotendinous structure [19]. To observe the relationship between the abduction/adduction of the CMC and MCP joint, comparison of two angles were conducted. Actual angles of the abduction/adduction at the CMC and MCP joint were calculated by MoCap data, while the thumb finger was induced to have a posture of clenching a fist. The abduction/adduction angle of MCP joint was assumed to be obtained linearly to the abduction/adduction angle of the CMC joint using least-squares, as follows:

(6) $$\begin{array}{ccc} \theta_{AA,MCP} & = & a_{1}\theta_{AA,CMC}+a_{2} \end{array}$$

where $a_{1}$ and $a_{2}$ were identified as 0.89 and 2.89 by the least square method. The RMS error was about 5.13 deg. The fitted and measured abduction/adduction angles of MCP joint are shown in Figure 7b.

**Figure 8:**
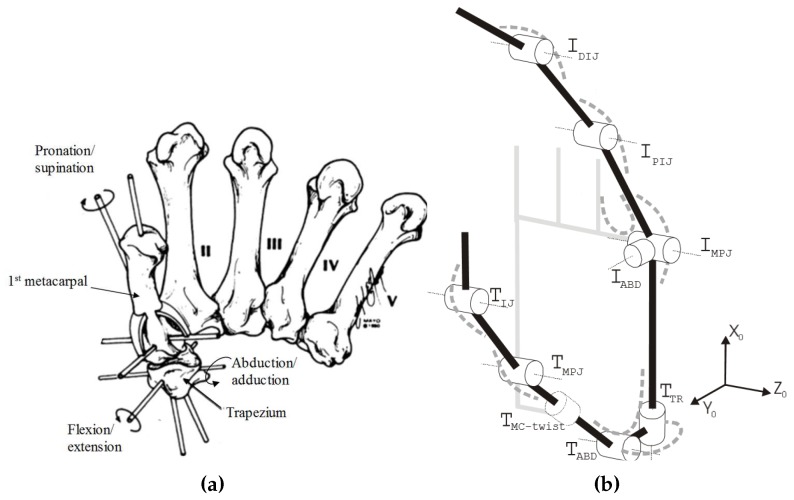
Examples of finger joint models; (**a**) Anatomical model for the CMC joint [18]; (**b**) finger joint model for the cyberglove [20].

The complex musculoskeletal structure of the CMC joint requires modeling analysis to measure the 3-D finger motion. Cooney et al. introduced three axes of rotation that are orthogonal each other with regard to the trapezium and the first metacarpal bones as shown in Figure 8a [18]. In general, the thumb CMC joint is a saddle joint with two DOFs between the trapezoid and the first metacarpal bone shown in Figure 8a. Although the saddle joint usually has two DOFs, it exhibits 3 different motion: the flexion/extension, abduction/adduction and axial rotation when ligaments of the bones are lax [18].

Although it has three axes of rotation, the axial rotation angle shows dependency on flexion and abduction. Griffin et al. introduced a model that the CMC joint has roll motion instead of the flexion/extension, separating each joint as revolute joints with offsets as shown in Figure 8b [20]. However, as the rotation (flexion/extension) motion is fixed to the palm, it is not suitable to express changing flexion/extension axis. Many offsets of the CMC joint, which are distances between the adjacent joints, (e.g., between $I_{ABD}$ and $T_{TR}$, $T_{TR}$ and $T_{ABD}$, $T_{ABD}$ and $T_{MC-twist}$ in Figure 8b), are included to the model, and the offsets are widely varied depending on the size of hand. Hollister et al. introduced a model which has non-intersecting and non-perpendicular axes of flexion/extension and abduction/adduction to express the dependent axial rotation [21]. However, as the angle between the two axes and the offset are widely variable for individuals, this model is not suitable for the proposed soft sensor system. Therefore, a model that can be applied to the different hand and optimized with the soft sensor based system was needed.

**Figure 9:**
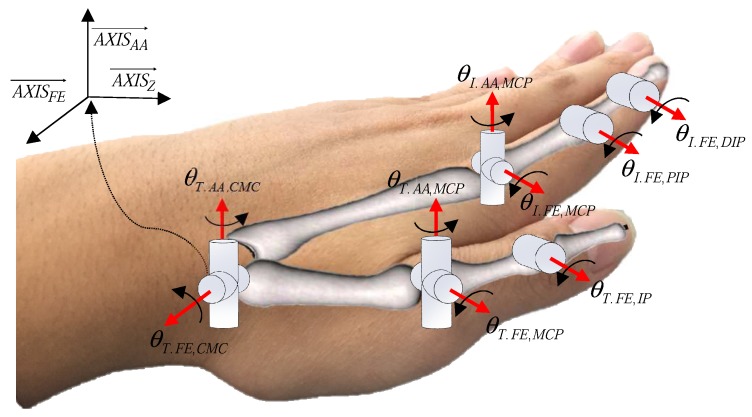
Proposed model for the finger joints.

The proposed model in this paper expresses the rotation axes as epidermal notation rather than the anatomic notation because the soft sensor was attached on the skin to measure the motion. The CMC joint was modeled to have two intersecting and perpendicular rotation axes with the flexion/extension and abduction/adduction, while keeping the orthogonality of the two axes [18]. As the amount of the axial rotation angle is relatively small (17 deg for the normal movement) and the limited space for the phalanges on thumb finger, axial rotation was neglected and the number of soft sensor was reduced [18,22,23]. The model for the CMC joint without the pronation rotation was suggested as shown in the Figure 9.

**Figure 10:**
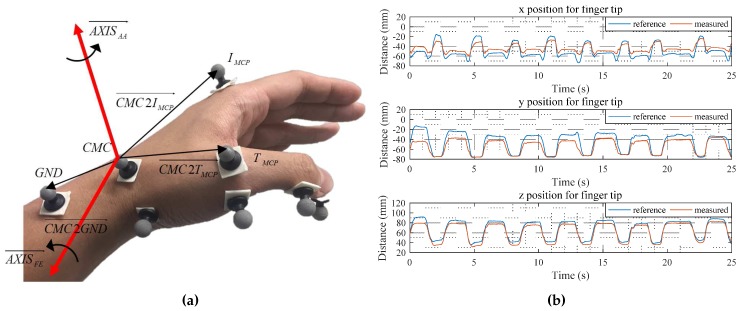
Experimental verification for the thumb finger model; (**a**) Defining rotation axes at the CMC joint; (**b**) Position of the thumb fingertip measured by the proposed model.

For the verification of the proposed thumb model, the position from the CMC joint to the fingertip calculated by the proposed model was compared with the MoCap data. The reference fingertip position was obtained from the MoCap data of the CMC joint to the fingertip. The position based on the proposed model was calculated from the initial position with the defined rotation axes and joint angles. The rotation axes were defined from the reflective marker data of the MoCap as shown in Figure 10a and they are $\vec{AXIS_{AA}}$ and $\vec{AXIS_{FE}}$, respectively, as follows:

(7) $$\begin{array}{ccc} \vec{AXIS_{AA}} & = & \vec{CMC2T_{MCP}}\times \vec{CMC2I_{MCP}} \end{array}$$

(8) $$\begin{array}{ccc} \vec{AXIS_{FE}} & = & \vec{CMC2T_{MCP}}\times \vec{AXIS_{AA}} \end{array}$$

The rotation axis for the abduction/adduction of the MCP joint was obtained parallel to the abduction/adduction of the CMC joint, and the axes for the flexion/extension of the MCP and IP joint were obtained parallel to the flexion/extension of the CMC joint. The joint angles were also calculated by the MoCap data. After calculating the vectors from the 3-D marker data, the angles between the vectors were calculated as shown in Figure 10b. Root-mean-square (RMS) error of the *x*,*y* and *z* positions was about 8.88 mm, 11.71 mm and 5.48 mm, respectively, which are in tolerable range for the finger motion measurement [4,5,6,7,24].

As a result, two Type 2 sensors were attached between the trapezium and first metacarpal and between first and second metacarpal to measure the flexion/extension and the abduction/aduction joint angles, respectively, as shown in Figure 3. As the MCP and IP joints of the thumb have a similar musculoskeletal structure with the PIP and MCP joint of the four fingers except the phalanges’ length, two Type 2 sensors were attached for the flexion/extension of the thumb MCP and IP joint.

## 4. Decoupling Algorithms for the Soft Sensor Signals

**Figure 11:**
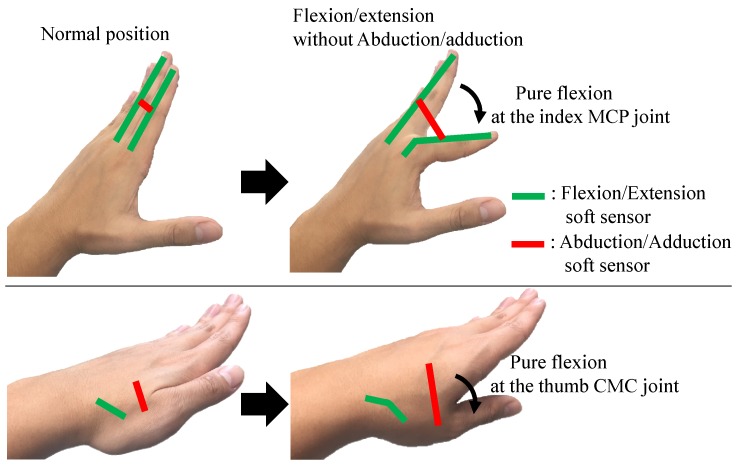
Necessity of algorithms decoupling the soft sensor signals at the CMC and MCP joints.

The flexion/extension angles of the MCP, PIP, and DIP joints of the four fingers were measured by Type 1 sensors and the abduction/adduction angles were measured by Type 2 sensors. However, the abduction/adduction sensors were elongated by pure flexion/extension motion as shown in Figure 11 due to the attached location of the sensors. Thus, it is necessary to decouple the two sensor signals to independently measure the flexion/extension and abduction/adduction angles. Since the CMC and MCP joints of the thumb have two perpendicular and intersecting rotation axes in the proposed model, similar decoupling algorithms can be applied to extract the pure flexion/extension and abduction/adduction joint angles.

**Figure 12:**
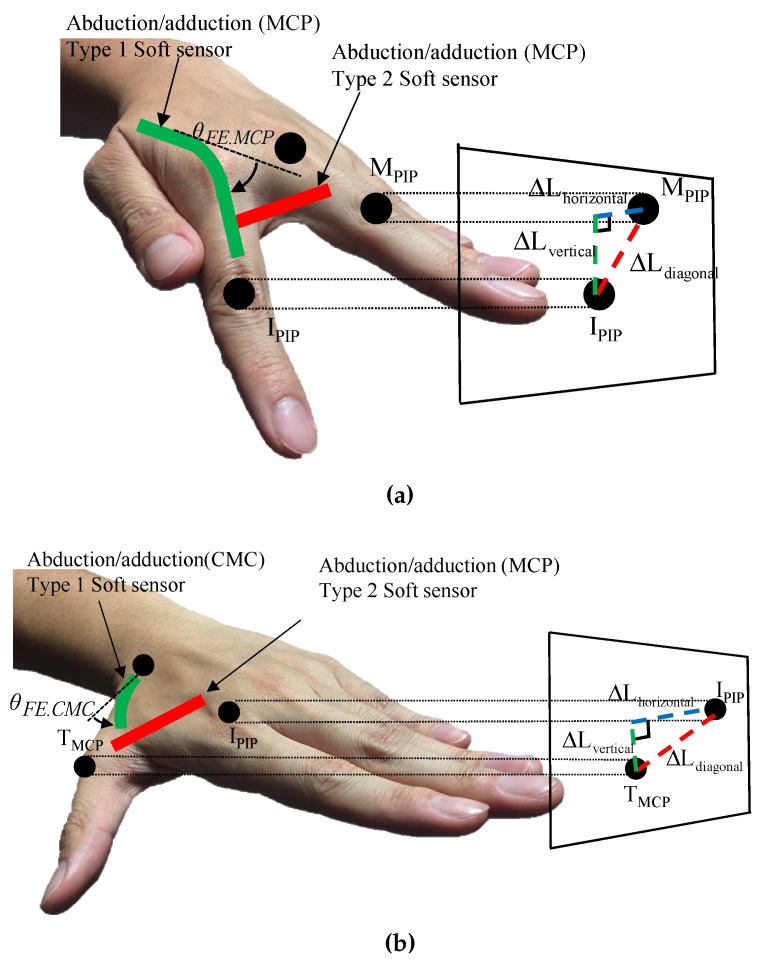
Decoupling algorithms for the sensor signals: (**a**) Decoupling algorithm for the MCP joint; (**b**) Decoupling algorithm for the CMC joint.

The soft sensor for the abduction/adduction measures $L_{diagonal}$ as shown in Figure 12a. The flexion/extension soft sensor was positioned on the axis such that any strain of the flexion/extension sensor was not affected by the strain of the abduction/adduction sensor when the MCP joint exerts a pure abduction/adduction motion. Therefore, the abduction/adduction sensor (Type 2 sensor) was placed 15 mm from the MCP joint of four fingers, and directly measured the linear displacement from the sensor as shown in Figure 3.

From the point of view shown in Figure 12a, the position of the PIP joint of the index ($I_{PIP}$) in 3-D can be expressed on the projected triangle formed by the position of middle finger’s MCP joint, $M_{MCP}$ and each length of the projected triangle has a relationship as below:

(9) $$\begin{array}{ccc} \Delta L_{horizontal} & = & \sqrt{\Delta L_{diagonal}^{2}-\Delta L_{vetical}^{2}} \end{array}$$

$\Delta L_{diagonal}$ was obtained from the abduction/adduction soft sensor and $\Delta L_{vertical}$ were linearly obtained by the measured flexion/extension angle with the sine function. Consequently, $\Delta L_{horizontal}$ was calculated as above relation (9) using linearly obtained $\Delta L_{diagonal}$ and $\Delta L_{vertical}$ from the abduction/adduction and flexion/extension soft sensor, respectively. The abduction/adduction angle in 3-D was calculated by linear mapping of $\Delta L_{horizontal}$ to the angle. Please note that exact length of proximal phalanx was not needed to extract the angles as in the joint radii cases because the strain of the sensor was directly mapped with the joint angles.

For the CMC joint of the thumb, as shown in Figure 11, sensor signals near the CMC joint also need to be decoupled with because the pure flexion/extension motion makes the abduction/adduction soft sensor deformed. As shown in Figure 12b, the triangle also was formed by the MCP joint of the thumb ($T_{MCP}$) and MCP joint of the index ($I_{MCP}$). $\Delta L_{diagonal}$ and $\Delta L_{vertical}$ were also obtained from the abduction/adduction and flexion/extension soft sensors at the CMC joint. Consequently, the calculated $\Delta L_{horizontal}$ was converted to the abduction/adduction angle.

## 5. Experimental Verification

**Figure 13:**
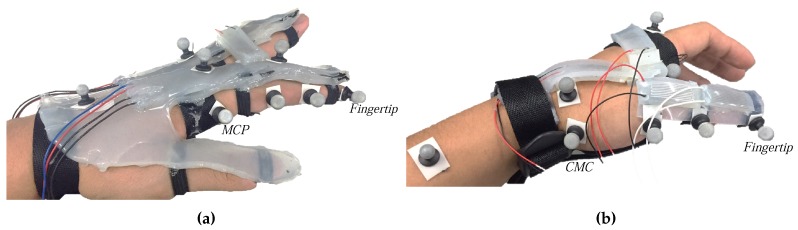
Experimental setup to verify the 3-D finger motion: (**a**) Attached markers on the index; (**b**) Attached markers on the thumb.

For the experimental verification of the proposed finger motion measurement system, joint angles of the thumb and index finger by applying the decoupling algorithms were compared with the reference angles from MoCap data. The reflective markers were attached as shown in Figure 13a,b. The reference joint angles were calculated from the MoCap data and the measured joint angles were obtained from the soft sensor-based system. As an experimental setup, the amplifier (LT1637, Lienar Technology, [25]) was connected to each microchannel to amplify the sensor siganl, and the amplified signal was read by a data acquisition board (PCIe-6353, National Instruments, [26]) as the voltage unit.

The subject was induced to move the index as a conical motion wearing the soft sensor-based motion measurement system for the index finger. For the thumb, the posture of clenching the fist was conducted while the subject wore the soft sensor-based thumb motion measurement system. As a result, the measured joint angles and the reference joint angles were compared.

As the soft sensor measures the strain by the applied voltages depending on the resistivity of the soft sensors, calibration procedures based on the pre-determined postures were needed to convert these strain into the joint angles. The measured angle was calculated as below:

(10) $$\begin{array}{ccc} \theta_{measured} & = & \frac{\varepsilon_{measured}-\varepsilon_{min}}{\varepsilon_{max}-\varepsilon_{min}}\left(\theta_{max}-\theta_{min}\right)+\theta_{min} \end{array}$$

where $\varepsilon_{min}$ and $\varepsilon_{max}$ are the minimum and maximum strain of the soft sensor, $\theta_{min}$ and $\theta_{max}$ are the minimum and maximum joint angles, which was obtained from the MoCap data, and $\varepsilon_{measured}$ is the strain of the sensor in real-time.

**Figure 14:**
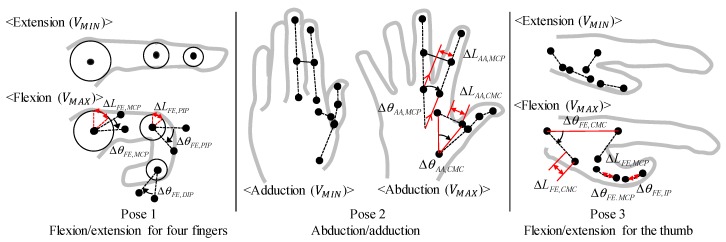
Calibration procedures.

For the index finger, the minimum and maximum angles of the flexion/extension of the MCP and PIP joint angles and the strain of the soft sensor were mapped using the pose 1 (Figure 14), and the DIP joint angle was proportionally calculated. The minimum and maximum abduction/adduction angles of the MCP joint and the strain of the sensor were mapped using the pose 2 (Figure 14). The minimum and maximum abduction/adduction angles of the CMC joint of the thumb and the strain were mapped using pose 2 (Figure 14). The minimum and maximum flexion/extension angles were obtained using pose 3 (Figure 14), and the flexion/extension angle of the MCP and IP joint was obtained similar with the calibration process of the index finger.

**Figure 15:**
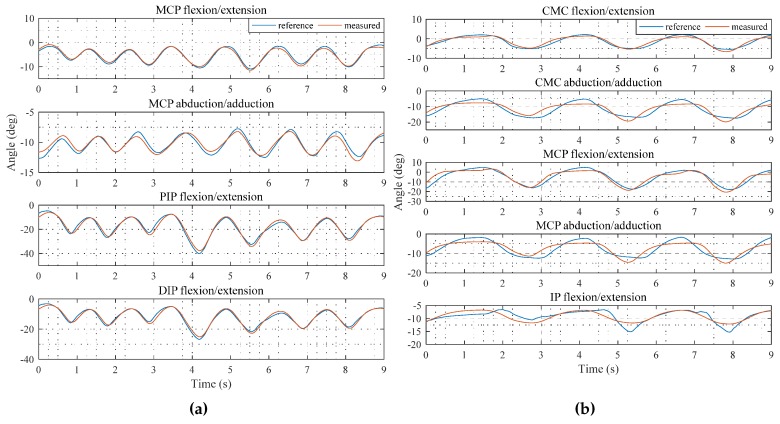
Comparison of the joint angles with MoCap data: (**a**) Comparison for the index finger; (**b**) Comparison for the thumb finger.

**Figure 16:**
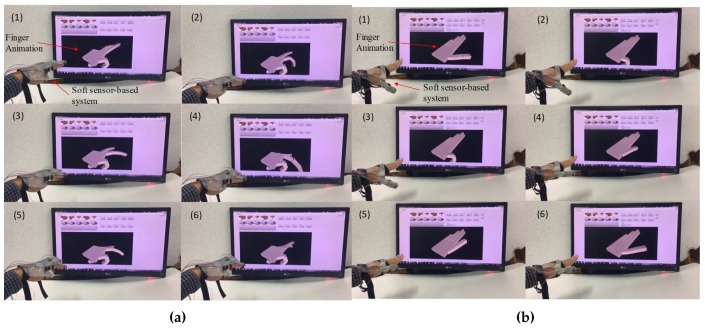
Finger animation by the measured 3-D finger joint angles: (**a**) Measured 3-D motion of the index; (**b**) Measured 3-D motion of the thumb.

The RMS error of the measured the flexion/extension and abduction/adduction of the MCP joint, the flexion/extension of the PIP and DIP joint for the index were about 0.66 deg, 1.44 deg, 0.96 deg, and 0.72 deg, respectively as shown in Figure 15a. For the thumb, the flexion/extension and abduction/adduction angles of the CMC, MCP and the flexion/extension angle of the IP joint were about 0.76 deg, 2.12 deg, 2.55 deg, 1.96 deg and 1.33 deg, respectively, as shown in Figure 15b. By using the joint angles, the animated 3-D finger motion was created as shown in Figure 16a,b. The animated 3-D motion was captured and summarized as 6 pictures for each motion.

As a result, the maximum and mean error for measuring joint angles was about 2.55 deg, and 1.39 deg, respectively. The main reason of the error might be the slip between the skin and the soft sensor. This could be reduced by tightly tied the fingers and soft sensors, but this may interrupt the natural finger motions. In addition, the slip between the soft sensors and reflective markers during the dynamic finger motions may be another plausible reason for the measurement error. In spite of these error factors, the performance of the finger motion measurement was accurate enough comparing with the other system [4,5,6,7,24].

In addition, regarding the virtual reality (VR) applications, the minimum mean variance of the joint angle for the various grasping pose in VR was mentioned as 0.1 rad (5.7 deg) [24]. Therefore, it is believed that 1.39 deg of mean error of the proposed system is tolerable enough to measure the motion of hand for VR applications.

## 6. Conclusions

A glove-based finger motion measurement system was developed using soft sensors made of microchannels molded silicone, and filled with the liquid metal (EGaIn). Owing to the superior elasticity of the silicone material, the soft sensor motion measurement system is easily wearable, light-weight, soft, and adaptable to the different hand size.

To simplify the motion of complex finger joints, a model of the CMC joint was proposed and verified by MoCap data. The soft sensor decoupling algorithms were designed and applied to the soft sensor-based system. As a result, 3-D finger motions at the CMC and MCP joint were measured by the soft sensor-based system and verified. The measurement error may be explained by the dislocation of the soft sensors from the skin, or the movement of the markers on the soft sensor it self. The viscoelastic properties of the silicone materials may cause an error when joints are moved quickly due to hysteresis effects.

In future work, the wearability of the sensors will be improved by reducing the displacement of markers on the skin and the sensor system. By using 3D printing technique for fabricating the soft sensors, such as embedded 3D printing (e-3DP) [8], the soft sensor-based glove will be fabricated consistently. A dynamic model will also be used to compensate for hysteresis caused by rapid motions [27]. These soft sensor-based systems could be applied to VR rehabilitation systems, which require accurate 3-D finger motion measurement [4,5,6,7,24].

## Figures and Tables

**Table 1:** Specification of Type 1 and 2 sensors.

	Type 1 Sensor	Type 2 Sensor
Sensor length [mm]	110	34
Sensor height [mm]	2	12
Sensor width [mm]	15	8
Channel length [mm]	810 (for the MCP)/930 (for the PIP)	395
Channel height [mm]	0.3	0.5
Channel width [mm]	0.3	0.5
